# Mental Health Needs and Services Utilization among Undergraduate and Graduate Students in Texas during the COVID-19 Pandemic

**DOI:** 10.3390/ijerph20126066

**Published:** 2023-06-06

**Authors:** Alice P. Villatoro, Vanessa L. Errisuriz, Melissa J. DuPont-Reyes

**Affiliations:** 1 Department of Public Health, College of Arts and Sciences, Santa Clara University, Santa Clara, CA 95053, USA; 2 Latino Research Institute, College of Liberal Arts and Sciences, University of Texas at Austin, Austin, TX 78712, USA; 3 Departments of Sociomedical Sciences and Epidemiology, Mailman School of Public Health, Columbia University, New York, NY 10027, USA

**Keywords:** COVID-19, higher education, college students, mental health, perceived need, mental health services

## Abstract

COVID-19 created a global crisis, exacerbating disparities in social determinants of health (SDOH) and mental health (MH). Research on pandemic-related MH and help-seeking is scarce, especially among high-risk populations such as college/university students. We examined self-rated MH and psychological distress, the perceived need for MH services/support, and the use of MH services across the SDOH among college/university students during the start of the pandemic. Data from the *COVID-19 Texas College Student Experiences Survey* (*n* = 746) include full- and part-time undergraduate/graduate students. Regressions examined self-rated MH, psychological distress, perceived need, and service use across SDOH, controlling for pre-pandemic MH, age, gender, and race/ethnicity. Economic stability was associated with higher risk of poor MH and need for MH services/support. Aspects of the social/community context protected student MH, especially among foreign-born students. Racial discrimination was associated with both greater psychological distress and use of services. Finally, beliefs related to the sufficiency of available institutional MH resources shaped perceived need for and use of services. Although the worst of the pandemic is behind us, the inequitable distribution of the SDOH among students is unwavering. Demand for MH support is high, requiring higher education institutions to better mobilize MH services to meet the needs of students from diverse social contexts.

## 1. Introduction

Alpha, Delta, and Omicron subvariants (e.g., XBB.1.5, XBB.1.16, XBB.1.9.1) have become familiar labels to describe the spread of COVID-19 across the globe. New coronavirus variants will continue to surface as we move from a pandemic to an endemic and fundamentally influence how our society responds to minimize COVID’s negative consequences on health, quality of life, and social mobility. Despite this global attention, there is much to be learned about the pandemic’s impact on mental health. Seasonal surges in COVID-19 infections have resulted in the increased promotion of effective mitigation strategies, such as vaccinations, mask wearing, and physical distancing. Likewise, individuals have had to learn to assess their individual risks for infection, severe illness, and death, redefining what it means to have a “normal life”. These situations may create stressors that impact individuals’ mental health, increasing the need for intervention and treatment. Student populations, especially those enrolled in higher education, underwent significant life changes because of the pandemic and may have experienced stressors that increased their risk of mental health problems [1,2,3]. To better prepare higher education institutions to support student mental health, it is vital to examine the impact of social, economic, and contextual factors in increasing the risk of poor mental health and promoting or impeding help seeking for mental health during the pandemic.

Psychiatric epidemiological evidence during the pandemic demonstrated growing mental health concerns among adult populations in the United States (US). Young adults (18–25) and socio-politically minoritized groups (e.g., race/ethnic minorities, sexual minorities) disproportionately experienced high levels of psychological distress, depression, and anxiety symptoms [4,5]. Among college students, similar escalations in internalizing behaviors, anxiety, depression, and psychological distress have been observed [1,2,3,6,7,8,9,10]. This evidence suggests an increasing need for mental health services during the pandemic, yet the use of these services among college students was low [11].

The mental health toll of the pandemic elicits concerns for academic outcomes of college populations. During lockdown, higher education institutions in the US transitioned from in-person to virtual classrooms. In the state of Texas, over 90% of courses in higher education institutions were face-to-face prior to the pandemic, but by fiscal year 2021 this figure had dramatically changed, with nearly 80% of students engaging in virtual rather than in-person learning [12]. With shifts in learning modalities, signs of changes in educational outcomes among college students emerged. For example, during the beginning of the pandemic, Latinx students in Texas from low-income backgrounds reported receiving lower grade point averages (GPA) and experiencing decreased motivation to complete coursework than those from high-income backgrounds [13]. The long-term implications of the COVID-19 pandemic on academic performance and post-graduate success are yet to be determined. It is plausible that deteriorating mental health observed among college students during the pandemic may have added to the burden of poor academic outcomes. There is prior research demonstrating how poor mental health can negatively affect GPA, retention, and graduation rates [14,15,16]. Thus, mental illness-related consequences of the pandemic may worsen future academic performance of students in higher education and impede social mobility later in life.

Burgeoning evidence of the rising mental health crisis during the COVID-19 pandemic has raised the alarm for public health to act. Yet, little is known about what factors increased the risk of these psychiatric conditions during the pandemic, especially for college populations. Moreover, mental health help-seeking behaviors, including actual use, avoidance, and perceptions of need for formal and informal support services, have not been well studied in a diverse population of college students. The current study examines patterns of mental health needs and the use of mental health services across the social determinants of health (SDOH) among undergraduate and graduate students enrolled in higher education institutions in Texas during the initial months of the pandemic.

### 1.1. Social Determinants of Health

The SDOH framework describes the root causes of health disparities, including disparities in mental illness and treatment use among socially disadvantaged populations [17]. According to this framework, the causes of health disparities relate to differential access to resources associated with (1) economic stability, (2) education access and quality, (3) healthcare access and quality, (4) neighborhood and built environment, and (5) social and community context. Together, the SDOH shape the environments and conditions that people are exposed to daily, affecting their access to resources and opportunities that impact health and wellbeing [18]. The use of this framework has provided extensive evidence of the impact of the SDOH on mental health [17,19,20]. The pandemic further widened inequities in resource distribution among socially disadvantaged groups, including college students [15,21]. It is imperative to understand how exposure to the SDOH during the pandemic shaped the mental health experiences of college students because it could inform the development of interventions, programs, and policies for preventing mental illness and promoting proactive mental health help seeking within institutions of higher learning. In the current study, we use the SDOH as a guiding theoretical framework for examining the mental health needs and service use patterns of college students in Texas during the pandemic.

### 1.2. Linking the SDOH of College Students to Mental Health during the Pandemic

The SDOH may have affected college students’ mental health and help-seeking behaviors during the pandemic in distinct ways. First, the pandemic increased economic uncertainty across the US. Unemployment increased, especially among young adults [22], likely intensifying financial hardship. Individuals who maintained employment throughout the pandemic, especially those who worked in frontline occupations where exposure to COVID-19 was high, may have experienced few employment protections (e.g., paid sick leave) [23]. For college students, school closures and relocation during lockdown also resulted in job loss, exacerbating financial instability, housing difficulties, and food insecurity [15,19,24,25]. Several studies have demonstrated how food insecurity experienced during the pandemic increased mental health burden (i.e., high psychological distress, depression, and anxiety) among US college students [19,26,27]. Another study found that college students who experienced high levels of housing instability and food insecurity during the pandemic were at increased risk of anxiety/depression and needing mental health services [25]. Although not a college student sample, another study points to how pandemic-related consequences pertaining to economic strain were associated with clinically significant somatization and anxiety [28]. However, many emerging studies in this area have used small samples of college students or recruited students from one institution. Importantly, while economic factors are common barriers to mental health services for college students [29], research on the economic consequences of the pandemic on mental health help-seeking behaviors in these populations have yet to be investigated.

Second, educational experiences fundamentally changed as institutions of higher learning suddenly and almost universally closed their campuses, sending many students home to participate in virtual learning. Certain students may have been more adaptable to these changes to learning. For instance, undergraduate education in the US is defined by the college experience, typically described as students living on campus, sharing physical spaces and residences with peers, attending in-person classes, engaging in diverse social activities in and outside of school, and starting to develop independence from their families. School closures and frequently changing policies related to COVID-19 disrupted these experiences for undergraduate students for nearly two years. Likewise, the shift from in-person to virtual learning impacted undergraduate classroom experiences, particularly affecting marginalized and less technologically resourced students (e.g., those from low-income backgrounds) [13]. In contrast, while graduate students experienced the same closures, adapting to these changes in learning may have come easier to them as graduate students are generally older and in a different life stage than undergraduates. Online learning studies suggest that graduate students tend to be more intrinsically motivated than undergraduate students, reporting higher use of self-regulated learning strategies and levels of academic motivation [30]. Graduate students also may have more life experience in managing competing demands (e.g., school, employment) that would facilitate virtual learning [31]. Furthermore, exposure to online learning may be more commonplace for certain graduate students as online graduate programs and part-time enrollment were popular pre-pandemic and continue to grow in popularity [32]. The extent to which the pandemic differentially affected mental health and mental health help seeking among undergraduate versus graduate students has not been explored in detail.

Third, higher education institutions may buffer the negative impacts of the SDOH on students’ mental health as they represent unique built environments that expose students to on-campus healthcare-related resources, recreational facilities, and programmatic opportunities that promote healthy lifestyles, help-seeking behaviors, and wellbeing. It is unclear whether the shift in physical environment (e.g., living on campus to living at home) affected mental wellbeing and how students sought help for mental health concerns. Students living on campus have convenient access to gymnasiums and recreational facilities that make it more feasible to engage in vigorous physical activity, which has been shown to mitigate mental health risks for depression and suicidal behaviors [33,34]. A recent study showed that students engaged in physical activity during the pandemic as a coping strategy for poor mental health [8]. Additionally, on-campus counseling and psychological services make mental health care more accessible to students, yet access to these on-campus services was disrupted by university closures and the limited supply of providers due to rising mental health demands [35]. A recent national study of US adults shows that despite the growing incidence of mental health problems during the pandemic, the use of treatment services remains low, with disparities in service use hurting vulnerable populations [36]. Of the limited published research, studies with small samples of college students have shown that the use of formal and informal mental health supports during the pandemic has been relatively low [37], especially among first-generation college students [38]. 

Finally, not all students experienced the pandemic equally as their social contexts shifted. International students, for example, faced uncertainty in their educational attainment trajectory. Transitioning from classrooms to virtual spaces introduced new issues about the eligibility of international students to continue to stay within the US [39]. In July 2020, the Trump administration introduced a policy that would have banned international students from staying in the US if all their classes were held online [40]. While this policy was not realized, international students and students of color confronted and/or witnessed vast racial discrimination during this time [41,42]. The amalgamation of these negative experiences may have compounded stress and resulted in increased levels of anxiety and depression for these students [43]. No study to date has examined how the changing social/community context of students, especially immigrant students and students of color, affected their use of mental health services during the pandemic.

### 1.3. Study Purpose

The ripple effects of the pandemic on SDOH and mental health will continue to unfold for years to come. Previous research on COVID-19 and mental health among US college students has limited generalizability as these studies have relied on small sample sizes, recruited samples of students from a singular academic institution, examined the role of specific SDOH on mental health status only, or have not investigated mental health services utilization. To better support mental health prevention and treatment efforts among college students, we must further examine the toll of the pandemic on mental wellbeing among these students not only with respect to symptoms, but also considering the types of services and supports students perceive to need and use. Moreover, as the pandemic shifted the distribution of SDOH, it is vital to understand how various dimensions of the SDOH affected the mental health symptoms and help-seeking behaviors of college students. The purpose of the current study is to examine patterns of mental health need as measured by symptoms screeners, the extent of perceived need for mental health support, and the use of mental health services. We use the SDOH framework to guide our analysis as to how mental health outcomes and help-seeking behaviors were impacted by (1) economic stability, (2) education access and quality, (3) healthcare access and quality, (4) neighborhood and built environment, and (5) the social and community context of college students. The study provides new evidence on the impact of COVID-19 on mental health by using data from a diverse sample of undergraduate and graduate students in Texas from 27 higher education institutions across the state.

## 2. Materials and Methods

### 2.1. Data

Data are from the *COVID-19 Texas College Student Experiences Survey*, a statewide cross-sectional study of COVID-19′s effects on health, wellbeing, and educational experiences among undergraduate and graduate students in Texas. Participants were 18 years or older and enrolled at least part time at any Texas higher education institution (community college, four-year institution, etc.). Participants of the web-based survey were recruited using informational flyers shared with school administrators and faculty and advertisements posted on social media pages of higher education institutions, and participants were asked to share the survey with their social networks. The 30 min survey was anonymous and contained questions on various health- and education-related topics, including mental health, substance use, physical activity, food insecurity, and educational experiences.

Data were collected and managed using REDCap^®^, a secure web platform for online surveys [44,45] hosted at the University of Texas at Austin (UT Austin). At the beginning of the survey, participants specified their consent to complete the survey and were asked brief questions to determine their eligibility. Those who declined participation or did not meet eligibility criteria did not complete the survey. Fifty participants who consented to participate and completed the survey were randomly selected to receive a USD 20 e-gift card of their choice. The study was approved by the Institutional Review Board of UT Austin.

### 2.2. Sample

The diverse convenience sample resulted in 1063 students from 27 higher education institutions who gave consent to participate. Of these participants, 44.03% completed questions on mental health, perceived need, and service utilization (*n* = 468). Compared to participants with complete data, those with missing data were more likely to be foreign-born and identify as Black or Asian. The current study uses multiple imputed data (see Data Analysis section for details). On average, students were in their late twenties (*M* = 28.53, *SD* = 8.94; not shown), working towards a graduate degree (75.60%), predominately female (73.01%), US-born (80.29%), and came from relatively low-income households (i.e., 64.20% with less than USD 60,000 annual household income, which was the median income for a household in the state of Texas in 2020, see Table 1). Most students were enrolled full time (78.28%). As expected, there were some significant differences between graduate and undergraduate students in the sample: graduate students were older, more likely to be non-Latino White, foreign-born, enrolled in school part time, and less likely to be uninsured than undergraduate students.

**Table 1:** Descriptive statistics of sample demographic characteristics, social determinants of health, mental health (MH) status, and MH help-seeking (*COVID-19 Texas College Student Experiences Survey*, 2020).

	Total Sample (*n* = 746)	Undergraduate Students (*n* = 182)	Graduate Students (*n* = 564)	Undergraduate vs. Graduate
	M (SD) or %	M (SD) or %	M (SD) or %	*p*-Value
***Demographic Characteristics***				
Male	26.99%	24.40%	27.82%	0.368
Age in years				
18–24	44.64%	75.27%	34.75%	<0.001
25–39	41.96%	19.78%	49.11%	<0.001
40–49	8.85%	2.75%	10.82%	0.002
50 and over	4.56%	2.20%	5.32%	0.089
Self-reported race/ethnicity				
Non-Latinx White	41.42%	26.92%	46.10%	<0.001
Latinx	23.86%	46.70%	16.49%	<0.001
Black	5.09%	3.30%	5.67%	0.210
Asian	14.88%	6.04%	17.73%	<0.001
Other	14.75%	17.03%	14.00%	0.318
***Social Determinants of Health***				
<USD 60,000 household income	64.20%	65.89%	63.66%	0.606
Lost job	20.89%	26.90%	18.95%	0.029
Undergraduate student	24.40%	---	---	---
Part-time student status	21.72%	4.40%	27.30%	<0.001
No health insurance	17.16%	26.92%	14.01%	<0.001
School provided sufficient MH resources				
No	18.36%	20.88%	17.55%	0.314
Yes	38.20%	36.26%	38.83%	0.536
I do not know	43.43%	42.86%	43.62%	0.857
Moved during pandemic	18.63%	32.97%	14.01%	<0.001
Foreign-born	19.71%	9.89%	22.87%	<0.001
Level of perceived discrimination	0.19 (0.42)	0.21 (0.81)	0.19 (0.49)	0.430
***Mental Health***				
Pandemic-related fair/poor self-rated MH	54.70%	62.22%	52.27%	0.045
Change in self-rated MH during the pandemic				
Better or same as before	42.82%	41.25%	43.32%	0.673
Worse than before	57.18%	58.75%	56.68%	
Serious psychological distress	25.49%	24.79%	25.71%	0.823
***Perceived Need for MH Support***				
Professional services				
No need	40.48%	35.25%	42.16%	0.146
Yes, but inadequate	39.54%	52.59%	35.33%	<0.001
Yes, and complete	19.98%	12.15%	22.51%	0.011
Social services				
No	46.83%	38.35%	49.56%	0.023
Yes	53.17%	61.65%	50.44%	
***Past 3-Month MH Service Use***				
In-person visit	6.71%	4.62%	7.39%	0.300
Virtual visit	18.02%	19.41%	17.57%	0.660
Avoided visit	19.35%	19.85%	19.19%	0.864

### 2.3. Measures

#### 2.3.1. Mental Health Outcomes

Subjective and objective measures of mental health were included. First, participants reported on the perceived status of their overall mental health before March 2020 (i.e., pre-pandemic) and after March 2020 (i.e., pandemic-related; 1 = *Excellent* to 5 = *Poor*). Pre-pandemic and pandemic-related self-rated mental health values were then dichotomized (0 = *Excellent*, *Very Good, and Good*; 1 = *Fair or Poor*). Using the pre-pandemic and pandemic-related dichotomous variables, a separate variable was constructed that captured changes in self-rated mental health before and after March 2020 (0 = *Self-rated mental health improved or stayed the same*; 1 = *Self-rated mental health worsened*).

Second, mental health symptoms were assessed using the Kessler 6 (K6), a six-item measure of 30-day psychological distress [46]. Items examined the frequency of feeling nervous, hopeless, restless/fidgety, depressed, that everything is an effort, and worthless (0 = *None of the time* to 4 = *All of the time*). Items were summed to create a total score (range 0–24; α = 0.89), and scores above the validated cut-off point of 13 were categorized as individuals with serious psychological distress (0 = *No*; 1 = *Serious psychological distress*), which is highly correlated with clinical mental disorders [46].

#### 2.3.2. Mental Health Help-Seeking Needs and Behaviors

Two domains of mental health help seeking were assessed. First, perceived need for mental health care and support—that is, the belief that intervention and/or support is necessary to abate mental health concerns—was measured using an adapted version of the Perceived Need for Care Questionnaire (PNCQ) [47]. Participants were asked five questions about their perceived need for mental health information, medications, counseling, support for practical issues (e.g., housing, employment), and support with daily functioning (e.g., caring for oneself) in the past three months (0 = *No perceived need*; 1 = *Yes*, *but received no help*; 2 = *Yes*, *but received minimal help*; 3 = *Yes*, *and received completed help*). Exploratory factor analyses revealed two factors: perceived need for professional mental health services (three items) and social services (two items). Responses to items within each factor were combined and ranked as *No Perceived Need*, *Inadequate Perceived Need*, or *Completed Perceived Need*. Due to low variation, perceived need with minimal help was combined with perceived need that resulted in no help (i.e., *Inadequate Perceived Need*).

Second, participants reported whether in-person and/or virtual mental health services were utilized within the last three months (0 = *No*; 1 = *Yes*). As shelter-in-place orders were prominent at the time of the survey, participants also indicated whether they had purposely avoided seeking mental health support from a specialty and/or medical provider in the past three months because of the pandemic (0 = *No*; 1 = *Yes*).

#### 2.3.3. Social Determinants of Health

Students completed a series of questions that assessed several dimensions of the SDOH. For *economic stability*, participants indicated the level of household income at the time of the survey (0 = USD 60,000 *or more*; 1 = *Less than* USD 60,000). They also reported regarding whether they had lost a job during the initial months of the pandemic (0 = *No*; 1 = *Yes*). Participants provided additional context on their *educational access and quality*, including enrollment status (i.e., 0 = *Full time*; 1 = *Part time*) and student classification (i.e., 0 = *Graduate*; 1 = *Undergraduate*). Two variables assessed *healthcare access and quality* among college students: health insurance status (0 = *Any insurance*; 1 = *Uninsured*) and perceptions on the sufficiency of mental health resources offered by their institution during the pandemic (0 = *Insufficient*; 1 = *Sufficient*; 2 = *Unknown*). As a proxy measure for assessing changes in *neighborhood and built environment*, students reported whether they had moved back home with family because of school closures (0 = *No*; 1 = *Yes*). Finally, two measures about the *social and community context* were included. The first inquired about each student’s nativity status (0 = *US-born*; 1 = *Foreign-born*). The second assessed the level of race-based discrimination students experienced in nine settings since March 2020 (home, work, healthcare settings, etc.; 0 = *Never* to 3 = *Often*). Eight items were drawn from Krieger’s (1990) measure of racial discrimination [48], with a ninth item assessing discrimination on social media. Mean scores were calculated to create a discrimination scale (alpha = 0.85).

#### 2.3.4. Control Variables

All analyses controlled for age (*18–24 years*—reference group; *25–39 years*; *40–49 years*; *50 and over*), self-reported gender (*Female*—reference group; *Male*), and race/ethnicity (*Non-Latinx White*—reference group; *Latinx*; *Black*; *Asian*; *Other race/ethnicity*). All outcomes, except for changes in self-rated mental health status, controlled for pre-pandemic self-rated mental health. These variables were selected because they are associated with the SDOH and have strong correlations with mental health outcomes and help-seeking behaviors.

### 2.4. Data Analysis

The goal of our analysis was to assess how exposures to the SDOH during the pandemic were associated with mental health needs and services utilization among college students in Texas. A series of logistic regression models examined how each SDOH domain was related to the probability of (a) having poor self-rated mental health, (b) worsening self-rated mental health, (c) serious psychological distress, and (d) engaging in or (e) avoiding mental health help seeking. Logistic models first included the SDOH variables to assess their unique contributions to the outcomes. The covariates were then added to observe changes in the direction and significance of effects of the SDOH and determine whether the SDOH remain relevant to mental health need and help-seeking outcomes after potential confounders are considered. The mental health outcome models exclude one of the variables for healthcare access and quality (i.e., perception of sufficiency of school mental health resources). The logistic regression results reported in Table 2, Table 3 and Table 4 present the exponentiated coefficients of the models (i.e., odds ratios).

Multinomial logistic regressions were performed to examine associations of the SDOH with the perceived need for professional mental health and social services; ordered logistic regressions were not appropriate for this analysis because the models violated the proportional odds assumption. Multinomial logit versus generalized ordered logit models were preferred because these models would allow us to compare perceived need categories (i.e., perceived need with inadequate support and perceived need with completed support) to a reference category of choice (i.e., having no perceived need). Tests of the independence of irrelevant alternatives (IIA) assumption of multinomial models were conducted for each perceived need outcome. Results revealed that there was no statistically meaningful distinction between people who reported inadequate versus complete need for social services. As such, these categories were combined, and a logistic regression model was used to examine the relationship between the SDOH variables and perceived need for social services. Similar to the logistic regression models, we first assessed the impact of the SDOH on the outcomes and then introduced the covariates to examine confounding factors. For the multinomial regression model, we present the exponentiated coefficients (i.e., relative risk ratios, or RRRs) for the perceived need for professional services outcome (see Table 3). RRRs represent the risk of observing a specific outcome (e.g., inadequate perceived need for professional services) relative to having no perceived need for a particular SDOH variable. All analyses were performed using STATA/SE 17 [49] and adjusted for the potential clustering of students within schools.

Approximately 40% of respondents were missing one of the outcome measures, SDOH, or covariates. Most missing data come from the mental health and service use variables, as these questions were positioned in the last third of the web-based survey. There were significant differences in nativity status and race/ethnicity between participants with complete and missing data. Additionally, missing data patterns were carefully examined, and there was evidence consistent with observations missing at random. As a result, multiple imputation was conducted to address the missing data. The following variables were imputed: self-rated mental health, serious psychological distress, perceived need for professional and social services, mental health service use (in-person, virtual, and avoidance), household income, gender, and perceived discrimination. Other variables were not imputed because missing data were minimal. Using STATA/SE 17 [49], multiple chained procedures were used to impute 25 data sets. Imputed data were combined using Rubin’s rules [50]. Complete and imputed case analyses resulted in similar patterns of results. Only imputed analyses are presented.

## 3. Results

### 3.1. Mental Health Status during the Pandemic

Table 1 presents descriptive statistics for the total study sample stratified by student classification. As prior research on COVID-19 and mental health has focused on undergraduate populations, the current study highlights differences between undergraduate and graduate students. During the initial months of the pandemic, over half of Texas college students in the sample self-reported fair/poor mental health, with undergraduate students demonstrating higher prevalence than graduate students (Table 1). Since March 2020, 60% of students indicated that their mental health worsened. One quarter reported symptoms of serious psychological distress, with similar prevalence observed between undergraduate and graduate students.

Table 2 displays results from logistic regression models examining associations between the SDOH and mental health outcomes. Controlling for the covariates, two SDOH were significantly associated with fair/poor self-rated mental health: education access/quality and social/community context. Part-time and foreign-born students, relative to full-time and US-born students, were less likely to rate their mental health as fair/poor during the initial months of the pandemic. Low-income students had greater odds of fair/poor self-rated health than high-income students, although this association was only marginally significant. Only nativity was associated with worsening self-rated mental health from pre-pandemic to pandemic, with foreign-born vs. US-born students being less likely to report worsening mental health. With respect to serious psychological distress, economic stability and social/community context were statistically significant SDOH. Students from low-income households and those who lost a job during the pandemic had higher odds of serious psychological distress than students from high-income households and those who maintained their employment status, respectively. Race-based discrimination experienced during the pandemic was also associated with increased odds of serious psychological distress. Healthcare access/quality and the neighborhood/built environment were not correlated with any mental health outcome.

**Table 2:** Logistic regression models examining associations of the social determinants of health on subjective mental health status and serious psychological distress among Texas college students (*COVID-19 Texas College Student Experiences Survey*, 2020).

	Pandemic-Related Fair/Poor Self-Rated Mental Health (*n* = 746)		Worsened Self-Rated Mental Health (*n* = 746)		Serious Psychological Distress (*n* = 746)	
	OR	[95% CI]	OR	[95% CI]	OR	[95% CI]
***Economic Stability***						
Household income						
USD 60,000 or more	1.00		1.00		1.00	
Less than USD 60,000	1.53 ^⤉^	[0.96, 2.44]	1.41	[0.91, 2.19]	1.67 *	[1.04, 2.68]
Lost job						
No	1.00		1.00		1.00	
Yes	1.29	[0.85, 1.97]	1.42	[0.92, 2.18]	1.65 **	[1.17, 2.33]
***Education Access and Quality***						
Student classification						
Graduate student	1.00		1.00		1.00	
Undergraduate student	1.12	[0.70, 1.79]	1.07	[0.72, 1.58]	0.77	[0.51, 1.18]
Enrollment status						
Full-time student	1.00		1.00		1.00	
Part-time student	0.73 *	[0.54, 1.00]	0.84	[0.60, 1.17]	0.91	[0.62, 1.35]
***Healthcare Access and Quality***						
Health insurance						
Any insurance			1.00		1.00	
Uninsured	1.10	[0.70, 1.72]	0.76	[0.45, 1.27]	1.27	[0.82, 1.97]
***Neighborhood and Built Environment***						
Moved during pandemic	1.28	[0.87, 2.87]	1.22	[0.84, 1.77]	0.98	[0.49, 1.98]
***Social and Community Context***						
Nativity status						
US-born			1.00		1.00	
Foreign-born	0.44 ***	[0.29, 0.67]	0.55 **	[0.38, 0.80]	0.76	[0.47, 1.25]
Perceived discrimination	1.29	[0.89, 1.86]	0.82	[0.58, 1.15]	1.36 *	[1.01, 1.82]

NOTE: ^⤉^ *p* < 0.10; * *p* < 0.05; ** *p* < 0.01; *** *p* < 0.001. Models control for pre-COVID-19 self-rated mental health, student age, self-reported gender, and race/ethnicity.

### 3.2. Perceived Need for Professional and Social Services

The perceived need for professional and social services to support mental health concerns was generally high in the sample (71.0% perceived any need; not shown in Table 1). Over the previous three months, almost 60% perceived a need for professional services, while approximately 50% perceived a need for social services (Table 1). However, of those students who reported a perceived need for mental health services, most received inadequate levels of support in professional settings (39.5%) and/or social service settings (43.8%; not shown); this was especially true for undergraduate relative to graduate students.

Table 3 presents the results of multinomial logistic regressions examining associations between the SDOH and the perceived need for professional services, adjusting for covariates. Three SDOH domains were associated with an inadequate perceived need for professional services relative to the base outcome of no perceived need: education access/quality, healthcare access/quality, and social/community context. Part-time versus full-time students were less likely to have an inadequate perceived need relative to the base outcome. Students who were satisfied with the level of school mental health resources available during the pandemic were less likely to have an inadequate perceived need compared to those who were not. Interestingly, students who were unaware of school mental health resources also reported lower inadequate perceived need compared to those who did not believe there were sufficient resources available. Finally, students with higher versus low levels of perceived discrimination were nearly twice as likely to report an inadequate perceived need for professional services.

**Table 3:** Multinomial and logistic regression models examining associations of the social determinants of health on perceptions of professional and social services for a mental health concern among Texas college students (*COVID-19 Texas College Student Experiences Survey*, 2020).

	Perceived Need: Professional Services (*n* = 746)		Perceived Need: Social Services (*n* = 746)	
	RRR	95% CI	OR	95% CI
NO PERCEIVED NEED (Base Outcome)				
**INADEQUATE PERCEIVED NEED**				
***Economic Stability***				
Household income				
USD 60,000 or more	1.00		1.00	
Less than USD 60,000	1.25	[0.87, 1.79]	1.78 **	[1.21, 2.63]
Lost job				
No	1.00		1.00	
Yes	0.70	[0.44, 1.12]	1.86 ***	[1.30, 2.68]
***Education Access and Quality***				
Student classification				
Graduate student	1.00		1.00	
Undergraduate student	1.41	[0.85, 2.33]	1.25	[0.75, 2.08]
Enrollment status				
Full-time student	1.00		1.00	
Part-time student	0.55 **	[0.37, 0.80]	0.98	[0.68. 1.41]
***Healthcare Access and Quality***				
Health insurance				
Any insurance	1.00		1.00	
Uninsured	0.92	[0.55, 1.54]	0.81	[0.54, 1.20]
School provided sufficient MH resources				
No	1.00		1.00	
Yes	0.19 ***	[0.11, 0.32]	0.44 ***	[0.31, 0.63]
I do not know	0.50 **	[0.32, 0.79]	0.62 **	[0.45, 0.85]
***Neighborhood and Built Environment***				
Moved during pandemic	1.19	[0.77, 1.83]	1.13	[0.73, 1.77]
***Social and Community Context***				
Nativity status				
US-born	1.00		1.00	
Foreign-born	1.20	[0.73, 1.99]	1.03	[0.66, 1.60]
Perceived discrimination	1.92 **	[1.24, 2.97]	1.47 ^⤉^	[1.00, 2.16]
**COMPLETED PERCEIVED NEED**				
***Economic Stability***				
Household income				
USD 60,000 or more	1.00			
Less than USD 60,000	1.22	[0.71, 2.09]		
Lost job				
No	1.00			
Yes	0.63*	[0.40, 0.98]		
***Education Access and Quality***				
Student classification				
Graduate student	1.00			
Undergraduate student	0.69	[0.38, 1.26]		
Enrollment status				
Full-time student	1.00			
Part-time student	1.06	[0.48, 2.33]		
***Healthcare Access and Quality***				
Health insurance				
Any insurance	1.00			
Uninsured	0.55 ^⤉^	[0.30, 1.02]		
School provided sufficient MH resources				
No	1.00			
Yes	0.53 ^⤉^	[0.26, 1.09]		
I do not know	0.50 ^⤉^	[0.25, 1.00]		
***Neighborhood and Built Environment***				
Moved during pandemic	1.01	[0.44, 2.29]		
***Social and Community Context***				
Nativity status				
US-born	1.00			
Foreign-born	0.45 *	[0.22, 0.93]		
Perceived discrimination	1.36	[0.80, 2.30]		

NOTE: ^⤉^ *p* < 0.10; * *p* < 0.05; ** *p* < 0.01; *** *p* < 0.001. The base outcome for the multinomial regression model was having no perceived need. All models control for pre-COVID-19 self-rated mental health, age, self-reported gender, and race/ethnicity.

For those who reported a perceived need for professional services and felt their needs were met (completed perceived need), economic stability and social/community determinants were important SDOH domains. Compared to students with stable employment, those who lost a job during the pandemic were less likely to have a completed need for professional services relative to the base outcome of no perceived need. Foreign-born students, in contrast to US-born students, were less likely to report completed perceived need relative to no perceived need. No other SDOH were associated with perceived need for professional services; healthcare access/quality was only marginally significant.

Logistic regression models examined the associations between SDOH and the perceived need for social services (Table 3). Different patterns emerged when compared to perceived need for professional services. Economic stability was a strong predictor of perceived need for social services: those from low-income households or who lost a job during the pandemic were nearly twice as likely to perceive a need for social services than students from high-income households or those who experienced job stability. Healthcare access/quality also was relevant in that students who were satisfied with the level of mental health resources offered by their institution reported lower perceived need for social services than those who were dissatisfied. Students unfamiliar with school mental health resources were also less likely to perceive a need for social services compared to those who believed insufficient mental health resources were available at the time. Perceived discrimination was only marginally associated with higher odds of perceived need for social services.

### 3.3. Mental Health Service Utilization

In general, mental health service utilization was low among the sample of Texas college students (Table 1). Students were more likely to report having virtual (18.0%) rather than in-person (6.7%) mental health visits during the initial months of the pandemic. However, nearly one in five students reported that they purposely avoided seeking mental health services during this same time. These patterns were consistent across undergraduate and graduate students.

Table 4 presents the results of logistic regressions examining associations between the SDOH and mental health service use, adjusting for covariates. Four SDOH variables were significantly related to in-person mental health service use. Students who lost their job during the pandemic versus those who did not were two times more likely to use in-person services. Similarly, students who experienced higher versus lower levels of discrimination were more likely to use these services. In contrast, undergraduate students and those who believed their institution provided sufficient mental health resources were less likely to use in-person services. Only perceived discrimination was associated with increased odds of virtual mental health visits during the pandemic. No other SDOH domains were associated with virtual visits.

**Table 4:** Logistic regression models examining associations of the social determinants of health on mental health help-seeking behaviors among Texas college students (*COVID-19 Texas College Student Experiences Survey*, 2020).

	Mental Health Service: In-Person Visit (*n* = 746)		Mental Health Service: Virtual Visit (*n* = 746)		Avoided Mental Health Services (*n* = 746)	
	OR	95% CI	OR	95% CI	OR	95% CI
***Economic Stability***						
Household income						
USD 60,000 or more	1.00		1.00		1.00	
Less than USD 60,000	1.33	[0.66, 2.66]	1.04	[0.70, 1.53]	1.06	[0.51, 2.22]
Lost job						
No	1.00		1.00		1.00	
Yes	2.26 *	[1.11, 4.60]	0.79	[0.48, 1.30]	1.34	[0.91, 1.98]
***Education Access and Quality***						
Student classification						
Graduate student	1.00		1.00		1.00	
Undergraduate student	0.36 *	[0.11, 0.98]	1.04	[0.63, 1.74]	0.88	[0.54, 1.45]
Enrollment status						
Full-time student	1.00		1.00		1.00	
Part-time student	0.87	[0.37, 2.03]	0.60 ^⤉^	[0.34, 1.04]	0.86	[0.58, 1.26]
***Healthcare Access and Quality***						
Health insurance						
Any insurance	1.00		1.00		1.00	
Uninsured	0.84	[0.36, 1.96]	0.61	[0.33, 1.12]	0.73	[0.39, 1.37]
School provided sufficient MH resources						
No	1.00		1.00		1.00	
Yes	0.48 *	[0.36, 1.96]	0.71	[0.40, 1.26]	0.26 ***	[0.16, 0.42]
I do not know	0.35 *	[0.15, 0.80]	0.62	[0.32, 1.23]	0.34 ***	[0.21, 0.56]
***Neighborhood and Built Environment***						
Moved during pandemic	1.46	[0.67, 3.18]	1.13	[0.66, 1.93]	0.87	[0.43, 1.31]
***Social and Community Context***						
Nativity status						
US-born	1.00		1.00		1.00	
Foreign-born	0.86	[0.31, 2.37]	0.91	[0.47, 1.76]	1.10	[0.60, 1.64]
Perceived discrimination	2.73 **	[1.46, 5.09]	2.39 ***	[1.48, 3.86]	2.02 *	[0.87, 3.51]

NOTE: ^⤉^ *p* < 0.10; * *p* < 0.05; ** *p* < 0.01; *** *p* < 0.001. All models control for pre-COVID-19 self-rated mental health, age, self-reported gender, and race/ethnicity.

Avoidance of help seeking was also common in this sample, but only two SDOH domains were associated with avoidance: healthcare access/quality and social/community context. Specifically, a belief that their institution provided sufficient mental health resources resulted in lower odds of avoiding mental health services when compared to students that did not believe their school provided enough resources. A similar pattern was observed among students who were unaware of the services available. Finally, students reporting high versus low discrimination were more likely to avoid services.

## 4. Discussion

The COVID-19 pandemic extended consequences beyond physical health; it created ripple effects on the SDOH, mental health status, and help-seeking behaviors of Texas college students. The current study provides additional evidence on the mental health effects of the pandemic by examining diverse populations of undergraduate and graduate students in Texas. Overall, the inequitable distribution of the SDOH among student populations is still apparent, impacting mental health and help-seeking behaviors. Our findings demonstrate that the mental health of students during the pandemic depended on the circumstances of their lives—that is, their exposure to resources that promote health and wellbeing. Without addressing these SDOH head on, we cannot expect to make headway in improving the mental health consequences of the pandemic, especially considering the low supply of mental health providers [51] and the overall underutilization of mental health services [11,36].

### 4.1. Economic Stability and Social Context Matter to College Students

The most prominent SDOH relevant to college students’ mental health and help seeking during the pandemic were economic stability and social/community context. In our sample of Texas students, those coming from low-income versus high-income households and those who lost a job compared to those who did not experienced a higher risk of serious psychological distress and demonstrated higher odds of in-person help seeking for mental health concerns. This finding is similar to other studies that have utilized smaller samples or been conducted outside the US [28,52]. Students experiencing economic hardship were also more likely to perceive the need for social services such as housing aid or support with daily functioning. For these students, the economic uncertainties of the pandemic may have resulted in higher stress and worries about making ends meet.

With respect to social/community context, students experiencing race-based discrimination had increased odds of serious psychological distress; these experiences, in turn, were associated with higher odds of needing and using professional mental health services. Although most students, particularly undergraduates, left college campuses and moved back home with family during the COVID-19 lockdown, encounters with discrimination were still common, despite limited in-person social interactions. The Stress Process Model suggests that acute and chronic stressors such as financial uncertainty (e.g., being able to pay one’s bills, experiencing food insecurity) and discrimination increase mental health symptoms, thereby increasing the need for services [53]. With discrimination increasing towards minoritized groups during the pandemic, especially Asian hate [41], these negative experiences also spread to international students overall [43]. However, our findings suggest that foreign-born versus US-born students were protected against poor mental health. Moreover, although discrimination increased the risk of poor mental health and motivated help seeking, it also acted as a deterrent towards mental health services. More exploration is required to understand why some students experiencing discrimination avoided mental health services while others sought out services during the pandemic.

### 4.2. Education and Healthcare Access and Quality as Enablers to Help Seeking

Education access and quality had limited impact on the mental health and help-seeking behaviors of students. Overall, part-time relative to full-time students were less likely to report fair/poor mental health and indicate they had an inadequate perceived need for professional mental health services. There were no statistically significant differences in mental health status across student classification. Disparities in help-seeking behaviors still existed as undergraduate students demonstrated lower use of in-person mental health services than graduate students. To our knowledge, our study is one of a handful that have examined mental health across student classification and/or enrollment status; previous studies have primarily focused on undergraduate populations [1,6,8,9,10,11]. Future research should examine other aspects of education, such as changes in the quality of classroom instruction during the pandemic and how these changes may have impacted mental health. Of equal importance is the need to understand the long-term impact of the rise in mental health problems during the pandemic on student academic performance, retention, and graduation.

Healthcare access and quality shaped the mental health help-seeking behaviors of Texan college students. While having access to health insurance was not associated with help seeking, awareness of school-related mental health resources was critical. Students who believed their school provided sufficient mental health resources as opposed to those who did not were significantly less likely to perceive a need for professional and social services or use in-person mental health services. Perhaps students who believed their institution offered sufficient resources were also satisfied with the type of resources made available. It is unclear what type of resources institutions may have offered that decreased the need for seeking in-person mental health services, as these resources could range from creating online spaces for students to assemble (e.g., peer support groups) to expanding the availability of telehealth providers. The belief that the school provided sufficient mental health resources was also associated with lower odds of treatment avoidance. Depending on the type of resources made available by institutions, these resources may have served as a gateway to formal mental health services for some students. The results of this study demonstrate that improving the supply of mental health resources within schools would be important for increasing access to and use of these services for all students, thereby reducing the level of unmet need among vulnerable student populations. Unexpectedly, students who were unaware of school mental health resources demonstrated different utilization patterns than those who believed resources were insufficient. These students experienced lower use of in-person mental health services and expressed lower odds of perceiving a need for professional and/or social services for mental health concerns. Students unaware of school-related resources may, in general, have lower awareness of mental health services within and outside of schools, deterring their use of these services overall.

The only SDOH not associated with any mental health or help-seeking outcome was the neighborhood/built environment. The lack of statistically significant findings could be due to the limitations in measurement. Moving back home with family during the pandemic was used as a proxy for measuring changes in the students’ built environment. For some students, returning home may have offered a sense of relief or comfort. The transition home may have been associated with better quality housing owing to privacy, space to quarantine, supportive kin and social networks, etc. However, for other students, the return home may have caused additional stress and worry due to unstable and/or unsafe home environments (e.g., domestic violence, neighborhood safety, multi-generational households, etc.). This measurement did not capture information on the quality of the environment when returning home. There is a need for future studies that investigate how specific aspects of the neighborhood/built environment influenced (or not) mental health experiences during the pandemic.

### 4.3. Study Limitations

The study findings should be considered in the context of the following limitations. First, the data are cross-sectional, representing a very specific period during the initial months of the pandemic. The study was only able to capture the immediate impacts of the pandemic, but mental health status, perceived need, and help seeking may have changed as the pandemic progressed. Data were collected the summer before the start of the 2020–2021 academic term, when most institutions in Texas shifted to fully remote campuses. It would be important to investigate if the mental health concerns observed during the beginning of the pandemic improved, worsened, or remained consistent as the pandemic progressed. Second, the current study examined how individual measures representing the SDOH domains were associated with mental health and help-seeking outcomes among college students rather than estimating the collective contribution of each SDOH domain on these outcomes. There is a need for future studies that evaluate both significance tests of individual variables in relation to outcomes and measures of effect size (e.g., change in $R^{2}$), as these approaches can minimize Type I errors (i.e., false positives) and enrich our understanding of the role of each SDOH domain in mental health and help seeking [54]. Third, while students enrolled in any higher education institution in Texas were invited to participate, the study sample is not representative of the larger population of Texas students. During fall 2019, Texas students in higher education were 33.7% White, 39.2% Latinx, and 13.0% Black [55]; the current study includes overrepresentation of white students but under representation of students of color. While the study cannot generalize the experiences of students of color, the data still provide valuable information on the impacts of the pandemic on mental health and help seeking among students overall. Lastly, over 40% of the sample had missing data on at least one of the variables of interest, which could have affected the precision of the analyses. However, the analyses incorporated the use of multiple imputation techniques to reduce potential bias due to missing data. The resulting findings were similar to those observed in complete case analyses.

These limitations notwithstanding, the current study provides growing evidence on how the COVID-19 pandemic affected the mental health and help-seeking behaviors of college students. To our knowledge, this is the first study conducted within the US to include students from across multiple institutions within the same state. Likewise, our study incorporates the perspectives of both undergraduate and graduate students; the latter group are underrepresented in the existing literature.

## 5. Conclusions

In the US, the public health emergency status of the COVID-19 pandemic expired on 11 May 2023, showing signs that the pandemic is transitioning into an endemic phase. We have come a long way since March 2020: effective vaccinations and boosters have become readily available to the public, COVID-19 cases have declined, and hospitalizations and COVID-19 related deaths have significantly improved. Despite these optimistic patterns, many questions remain about the long-term impacts of the pandemic, including the burden on mental health and the distribution of the SDOH.

For college students, the return to campus, while welcomed, did not come without a price. As illustrated in the current study, mental health concerns among Texas college students were significant in the initial months of the pandemic; self-rated mental health was poor and nearly one quarter reported having serious psychological distress, mirroring similar results in national epidemiological studies [4,5]. Institutions of higher learning in Texas are underprepared and understaffed to address the mental health demands of students on campus [56]. Some universities, such as Texas A&M University, have promoted the use of new mobile apps to expand access to short-term, virtual services. In 2022, the University of Texas (UT) System announced that it would invest USD 16.5 million over the next five years to expand and improve the quality of mental health services for students across its multiple campuses [57]. These funds will go towards (1) establishing a mental health crisis line, (2) expanding telehealth services, (3) providing substance use, sexual assault, and safety training to students, (4) training faculty and staff to recognize and respond to student mental health concerns, and (5) expanding use of UT Austin’s mental health-oriented smartphone app that focuses on skills building and guided exercises. As these efforts are implemented, the UT System will evaluate their impact.

While attention to increasing access to mental health services bodes well for college students, the results from our study suggest that these actions alone are insufficient to address the mental health crisis. As we transition into a world where humans and COVID-19 co-exist for perpetuity, we need to build mental health infrastructures within and outside higher education institutions to better support the needs of students. However, it is just as important to intervene on the SDOH that predispose students to poor mental health outcomes, especially after the pandemic. How can institutions of higher learning better support students who are experiencing extreme financial hardship? What can these institutions do to prevent race-based discrimination within school settings? These are questions that should be at the forefront of our conversations when discussing and planning actions to promote student mental health and wellbeing. Mental health is more than just a consequence of the pandemic; mental health is also a function of where people live, work, and study.

## Data Availability

Data are available on request due to privacy restrictions.
